# Supplementary material for: Structure and mechanism of a mycobacterial isoniazid efflux pump MsRv1273c/72c with a degenerate nucleotide-binding site
Source: Nat Commun. 2025 Apr 28;16:3969. doi: 10.1038/s41467-025-59300-5 (PMC12038006; doi:10.1038/s41467-025-59300-5)
Supplement: Supplementary file 1 — Supplementary Information [file 41467_2025_59300_MOESM1_ESM.pdf]

Supplementary Information

Structure and mechanism of a mycobacterial isoniazid efflux pump *Ms*Rv1273c/72c with a degenerate nucleotide-binding site

Jing Yu[1,#], Yuhui Lan[1,#], Chen Zhu[1], Zhendong Chen[1], Junyi Pan[1], Yanfeng Shi[1,2], Lan Yang[1,2], Tianyu Hu[1], Yan Gao[1], Yao Zhao[2], Xiaobo Chen[1], Xiuna Yang[1], Shuihua Lu[2], Luke W. Guddat[3], Haitao Yang[1,*], Zihe Rao[1,2,4,5,6,*], Jun Li[1,*]

[1]Shanghai Institute for Advanced Immunochemical Studies and School of Life Science and Technology, ShanghaiTech University, Shanghai 201210, China

[2]National Clinical Research Center for Infectious Disease, Shenzhen Third People's Hospital, Shenzhen, 518112, China.

[3]School of Chemistry and Molecular Biosciences, The University of Queensland, Brisbane, QLD 4072, Australia

[4]State Key Laboratory of Medicinal Chemical Biology, Nankai University, Tianjin 300353, China

[5]Laboratory of Structural Biology, Tsinghua University, Beijing 100084, China

[6]Innovative Center for Pathogen Research, Guangzhou Laboratory, Guangzhou 510005, China

[#]These authors contributed equally: Jing Yu, Yuhui Lan.

[*]Correspondence: yanght@shanghaitech.edu.cn (H.Y.), raozh@mail.tsinghua.edu.cn (Z.R.), lijun1@shanghaitech.edu.cn (J.L.)

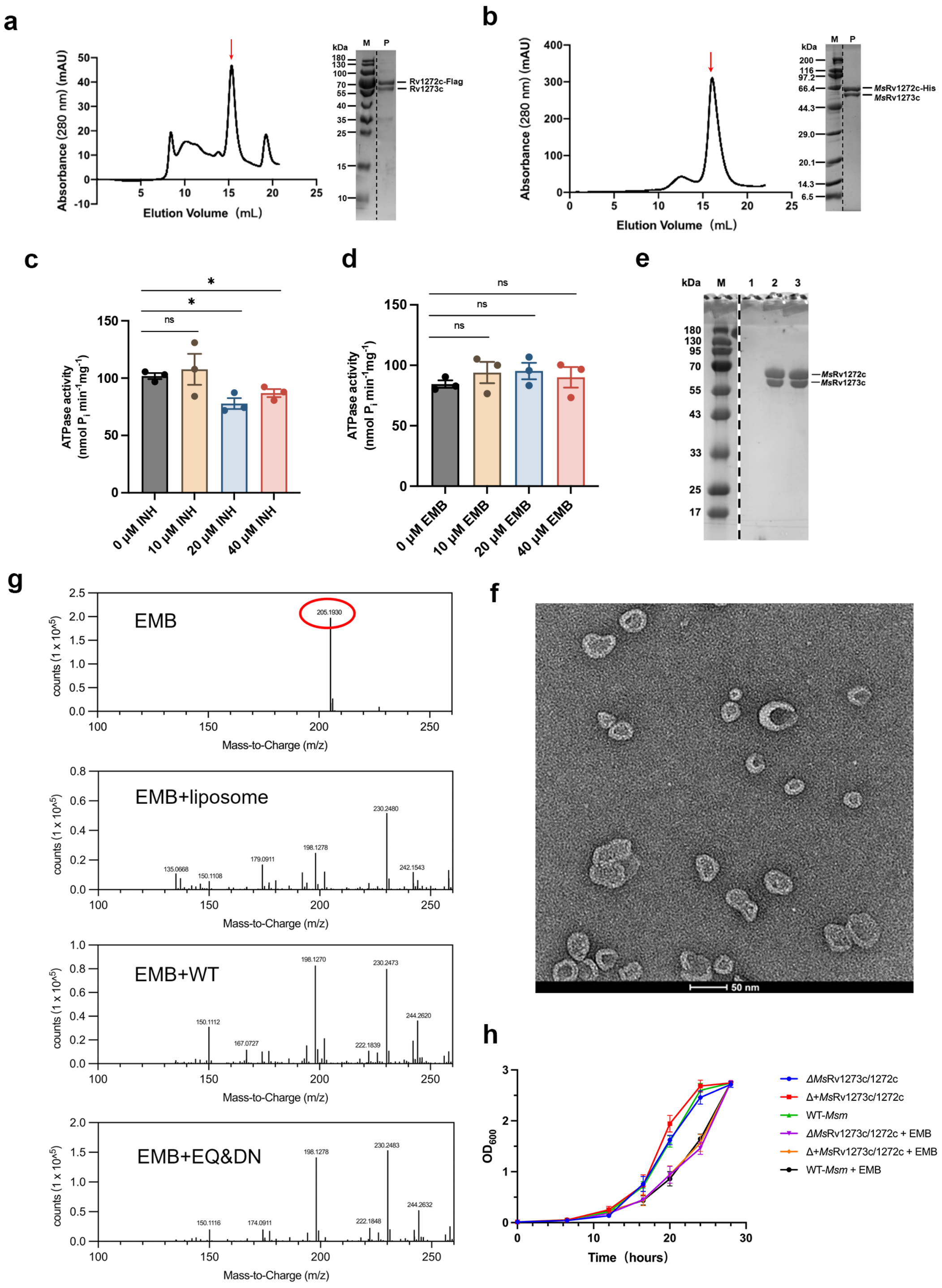

Supplementary Fig. 1 Purification and characterization of *Ms*Rv1273c/72c.

a Superose 6 gel filtration column chromatography (GE healthcare) for the Rv1273c/72c complex and the SDS-PAGE of the eluted peak indicated by the red arrow. M, molecular weight marker; P, peak elution. **b** Superose 6 gel filtration column chromatography for the *Ms*Rv1273c/72c complex and the SDS-PAGE of the eluted peak indicated by the red arrow. **c** The ATPase activity of *Ms*Rv1273c/72c when treated

with different concentrations of isoniazid (INH). Data are represented as the mean ± S.E.M., calculated from three biologically independent experiments (n = 3). *P* values were calculated using an unpaired two-sided t-test. ns, not significant; *, $P < 0.05$. 0 μM INH vs 10 μM INH, $P = 0.69$; 0 μM INH vs 20 μM INH, $P = 0.01$; 0 μM INH vs 40 μM INH, $P = 0.03$. **d** The ATPase activity of *Ms*Rv1273c/72c when treated with different concentrations of ethambutol (EMB). Data are represented as the mean ± S.E.M., calculated from three biologically independent experiments (n = 3). *P* values were calculated using an unpaired two-sided t-test. ns, not significant. 0 μM EMB vs 10 μM EMB, $P = 0.37$; 0 μM EMB vs 20 μM EMB, $P = 0.22$; 0 μM EMB vs 40 μM EMB, $P = 0.57$. **e** SDS-PAGE for the incorporation of proteins into liposomes by comparing the liposomes (lane 1) and proteins (lane 3) before reconstitution and proteolipsomes (lane 2) after reconstitution (sampled in the same volume). The bands corresponding to *Ms*Rv1273c and *Ms*Rv1272c in each lane were integrated to calculate the efficiency of reconstitution. **f** A representative image of negative stain EM for the proteolipsomes after reconstitution. **g** Mass spectrometry was used to determine the contents inside the liposomes with wildtype *Ms*Rv1273c/72c (WT), or with the E553Q/D497N double mutant (EQ&DN) inserted, and liposomes without any protein added. Pure ethambutol (EMB) was also measured as a standard. The red circle indicates the expected mass/charge of the drug. **h** Growth curves of wild type *Msm* (WT-*Msm*), *Ms*Rv1273c/72c knockout strain (Δ*Ms*Rv1273c/72c), and complemented strain containing pMV261-*Ms*Rv1273c/72c (Δ+*Ms*Rv1273c/72c) in the presence or absence of ethambutol. Data are presented as the mean ± S.E.M., calculated from three biologically independent experiments (n = 3).

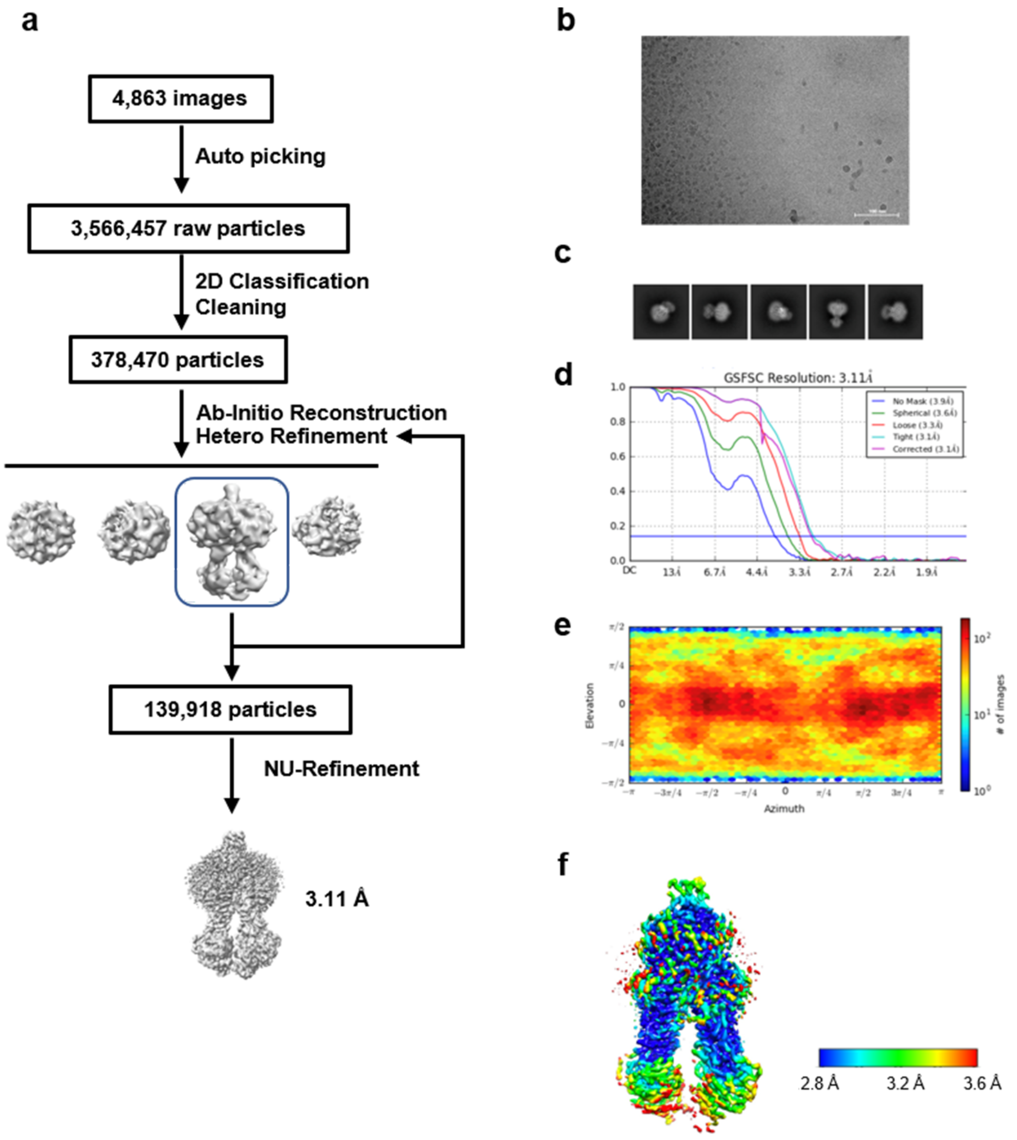

Supplementary Fig. 2 Cryo-EM data processing of *Ms*Rv1273c/72c in the IF^{apo} state.

a Flow chart for the processing of the cryo-EM data. **b** Representative cryo-EM micrograph. **c** Selected reference-free 2D class averages. **d** Gold-standard Fourier correlation curves of 3D reconstructions. **e** Posterior precision directional distributions of all particles used in the final 3D reconstruction generated by cryoSPARC. **f** The density map colored according to the local resolution estimation using cryoSPARC.

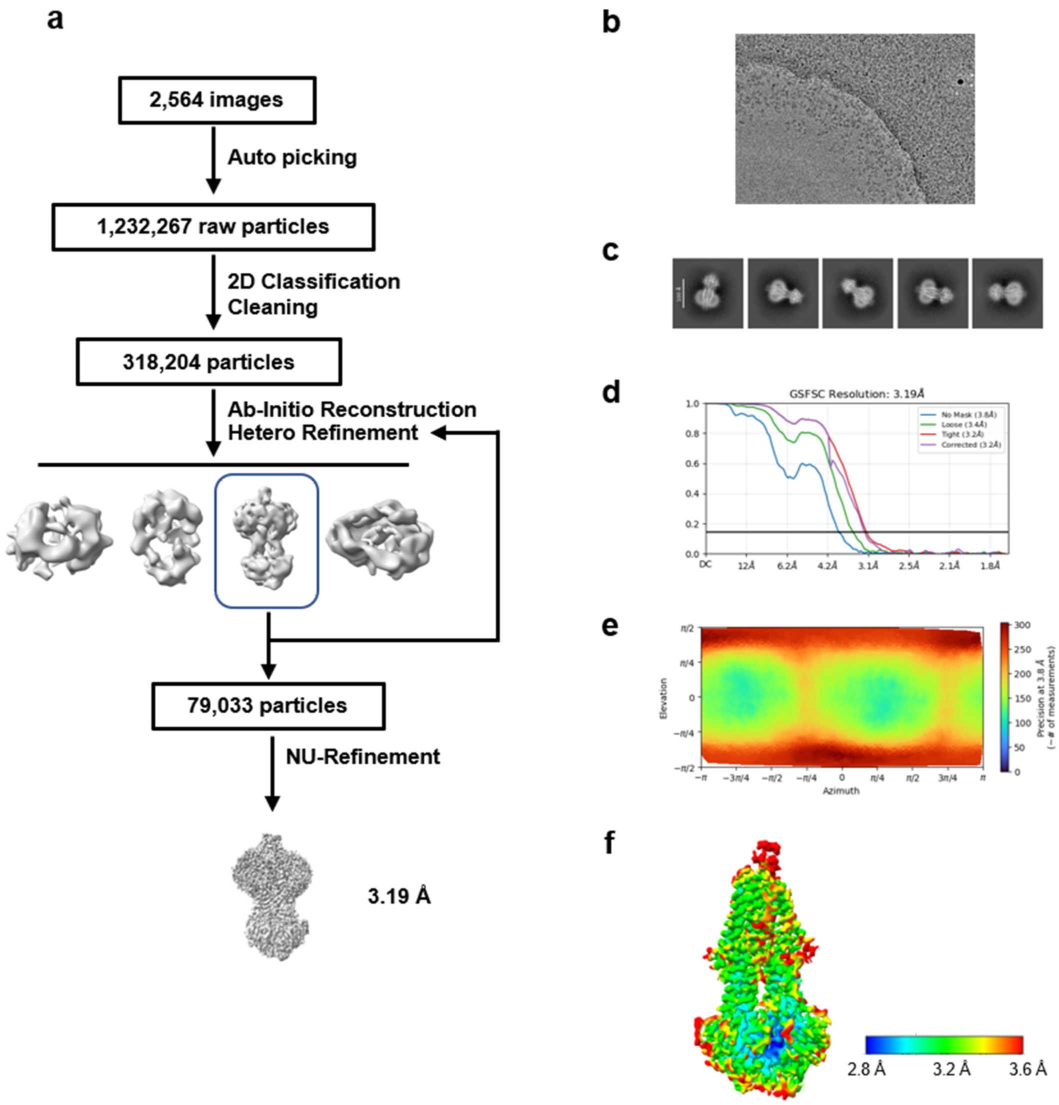

Supplementary Fig. 3 Cryo-EM data processing of *Ms*Rv1273c/72c^{E553Q} mutant in the ATP-bound Occ state.

a Flow chart for the processing of the cryo-EM data. **b** Representative cryo-EM micrograph. **c** Selected reference-free 2D class averages. **d** Gold-standard Fourier correlation curves of 3D reconstructions. **e** Posterior precision directional distributions of all particles used in the final 3D reconstruction generated by cryoSPARC. **f** The density map colored according to the local resolution estimation using cryoSPARC.

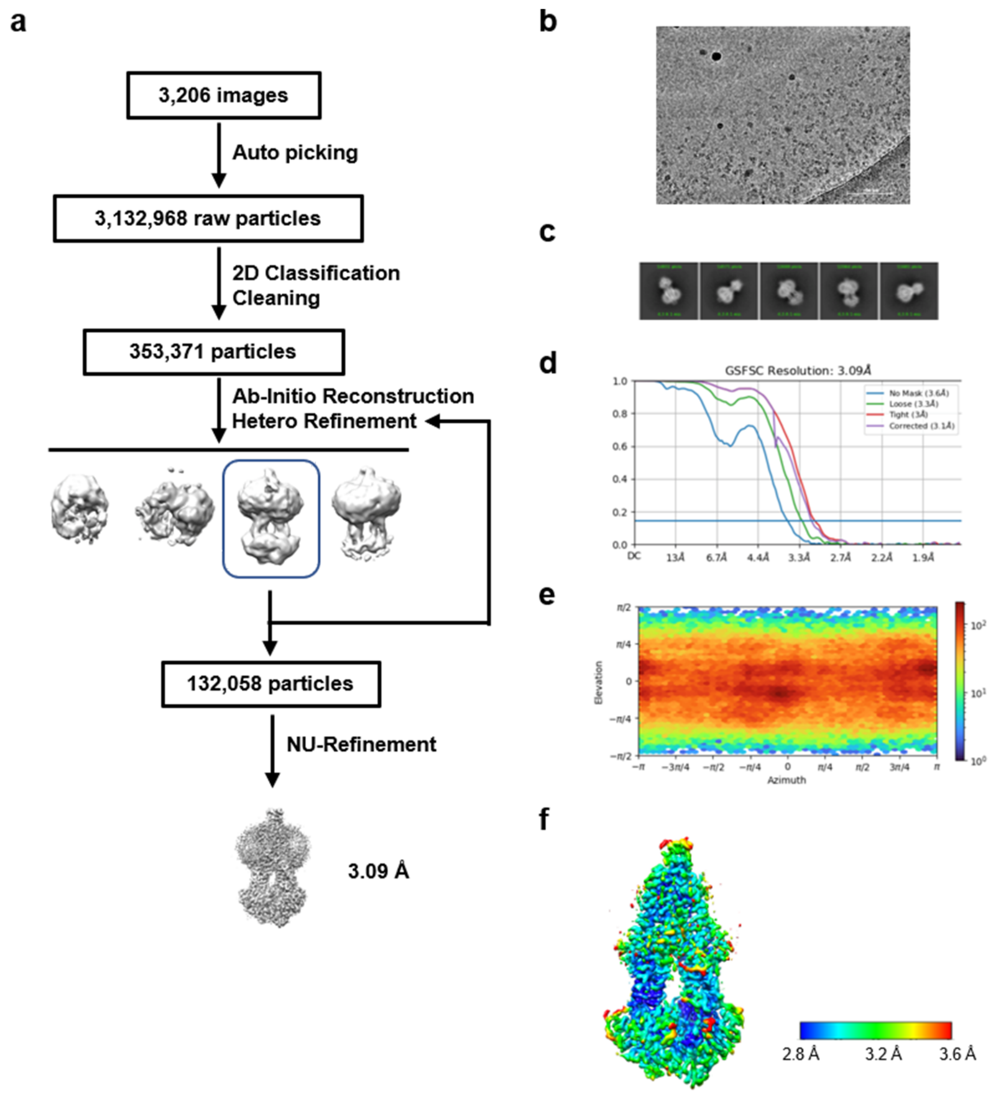

Supplementary Fig. 4 Cryo-EM data processing of *Ms*Rv1273c/72c in the AMPPNP-bound IF^{asym-1} state.

a Flow chart for the processing of the cryo-EM data. **b** Representative cryo-EM micrograph. **c** Selected reference-free 2D class averages. **d** Gold-standard Fourier correlation curves of 3D reconstructions. **e** Posterior precision directional distributions of all particles used in the final 3D reconstruction generated by cryoSPARC. **f** The density map colored according to the local resolution estimation using cryoSPARC.

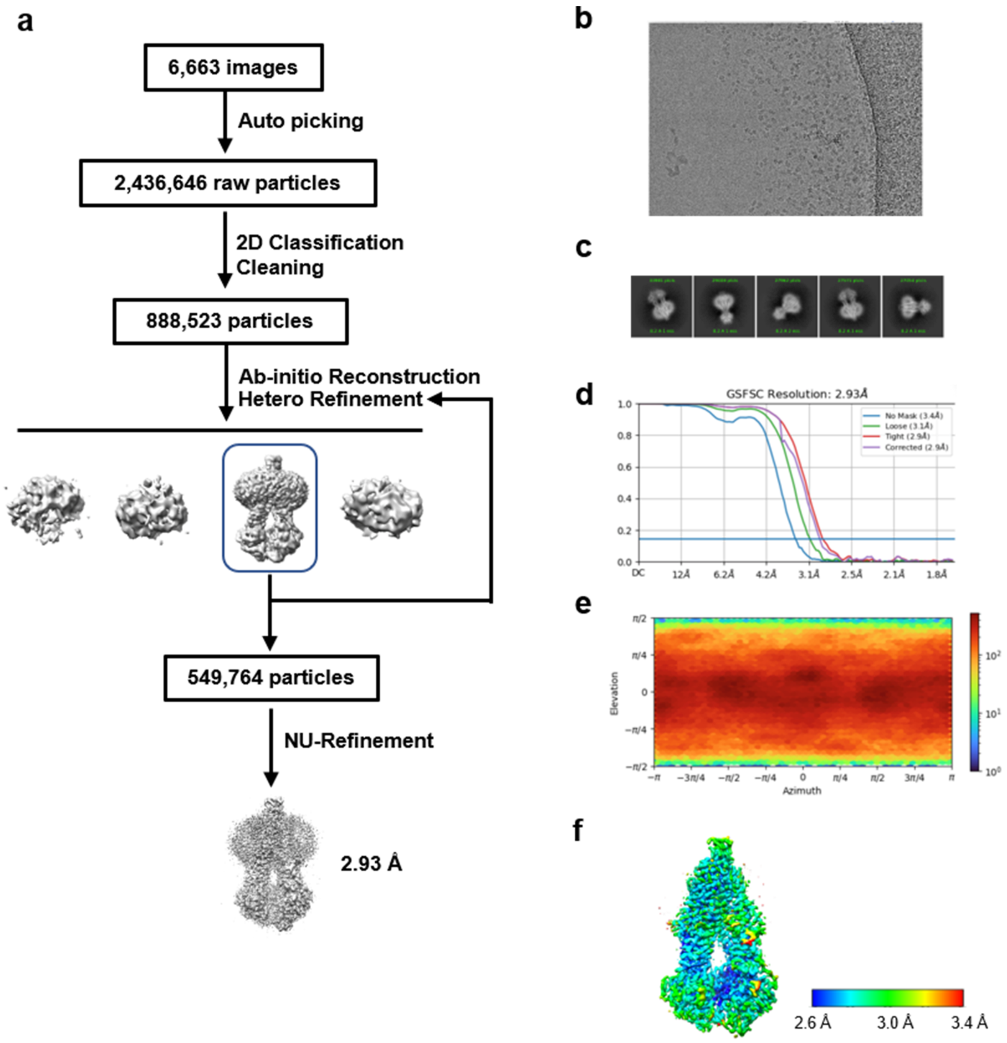

Supplementary Fig. 5 Cryo-EM data processing of *Ms*Rv1273c/72c in the ATP|ADP-bound IF^{asym-2} state.

a Flow chart for the processing of the cryo-EM data. **b** Representative cryo-EM micrograph. **c** Selected reference-free 2D class averages. **d** Gold-standard Fourier correlation curves of 3D reconstructions. **e** Posterior precision directional distributions of all particles used in the final 3D reconstruction generated by cryoSPARC. **f** The density map colored according to the local resolution estimation using cryoSPARC.

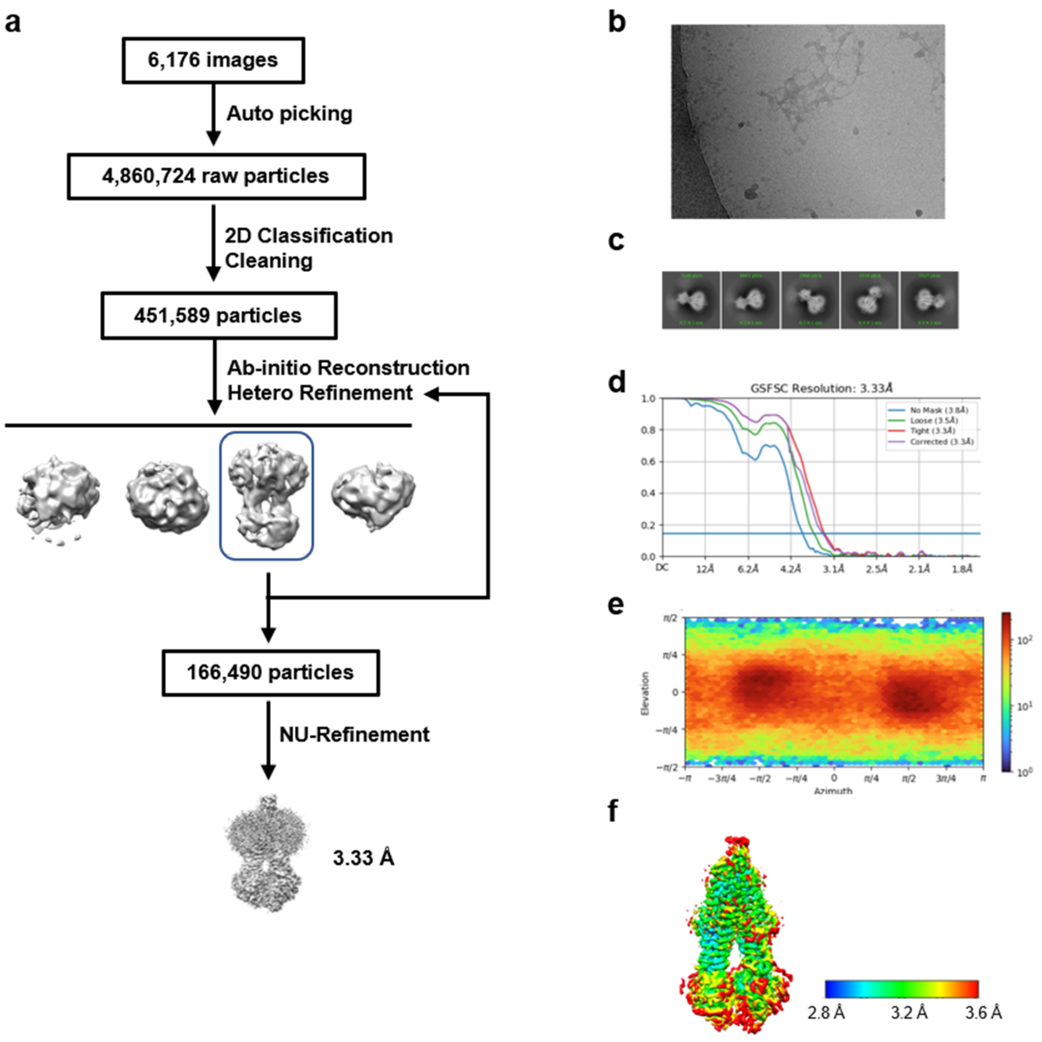

Supplementary Fig. 6 Cryo-EM data processing of *Ms*Rv1273c/72c in the ADP-bound IF^{asym-3} state (ATP 37 °C).

a Flow chart for the processing of the cryo-EM data. **b** Representative cryo-EM micrograph. **c** Selected reference-free 2D class averages. **d** Gold-standard Fourier correlation curves of 3D reconstructions. **e** Posterior precision directional distributions of all particles used in the final 3D reconstruction generated by cryoSPARC. **f** The density map colored according to the local resolution estimation using cryoSPARC.

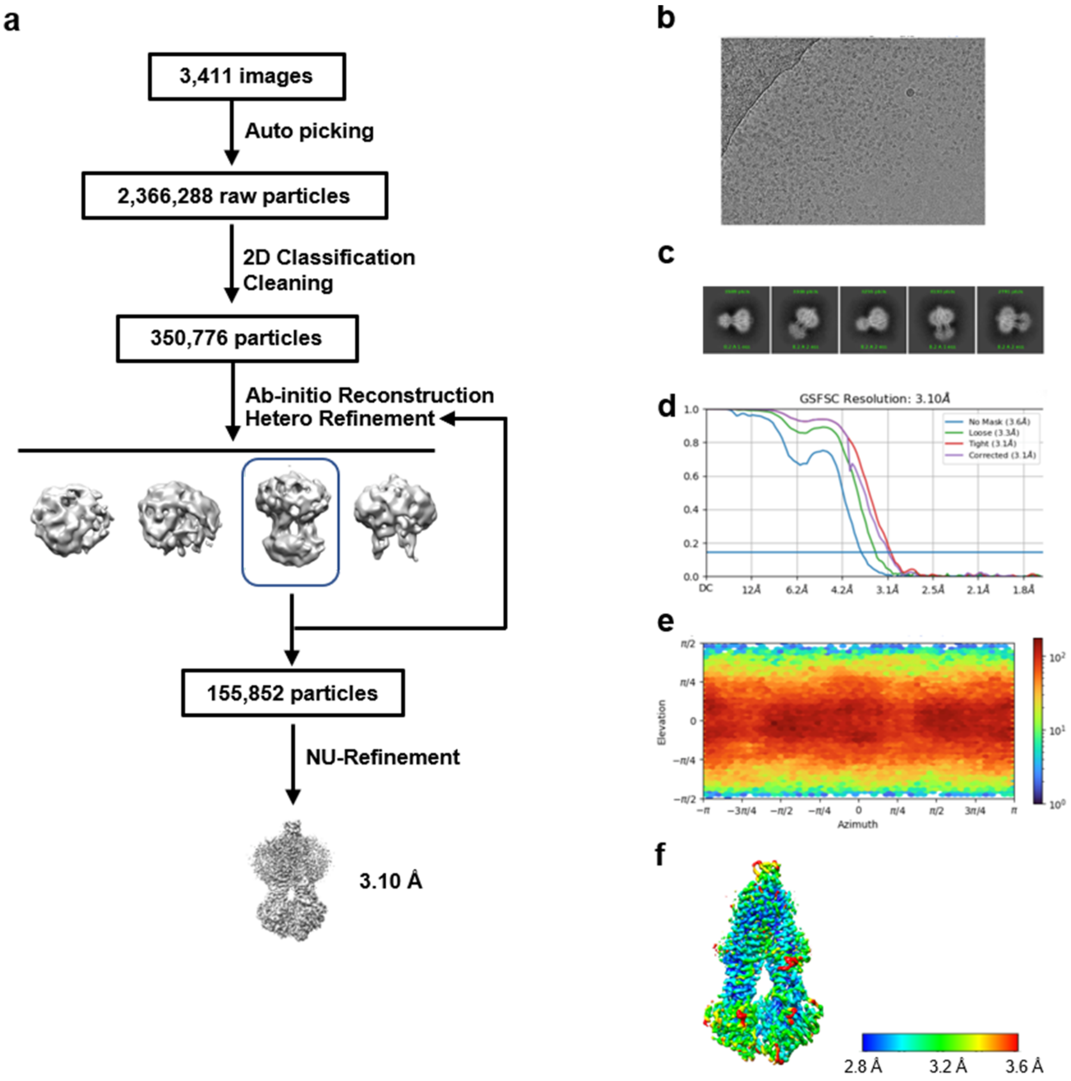

Supplementary Fig. 7 Cryo-EM data processing of *Ms*Rv1273c/72c in the ADP-bound IF^{asym-3} state (ADP 4 °C).

a Flow chart for the processing of the cryo-EM data. **b** Representative cryo-EM micrograph. **c** Selected reference-free 2D class averages. **d** Gold-standard Fourier correlation curves of 3D reconstructions. **e** Posterior precision directional distributions of all particles used in the final 3D reconstruction generated by cryoSPARC. **f** The density map colored according to the local resolution estimation using cryoSPARC.

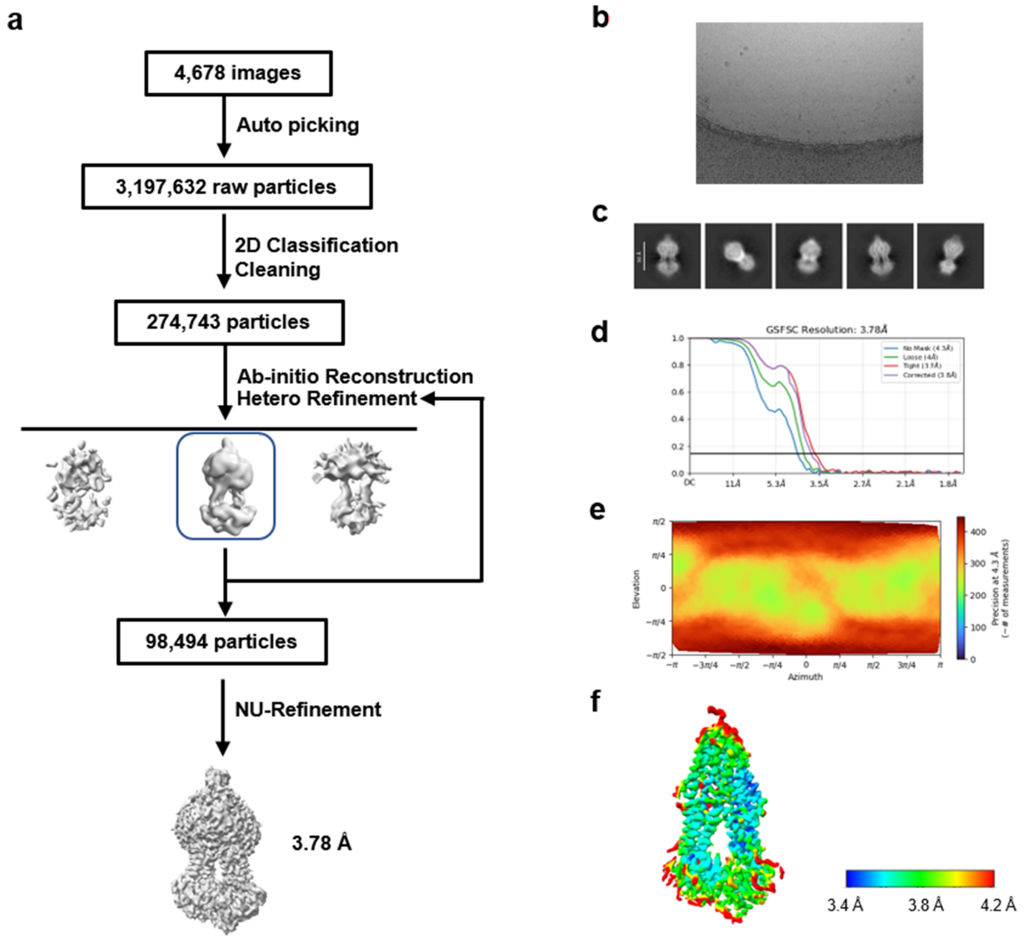

Supplementary Fig. 8 Cryo-EM data processing of *Ms*Rv1273c/72c in the ADP-bound IF^{asym-3} (peptidisc) state (ATP 37 °C).

a Flow chart for the processing of the cryo-EM data. **b** Representative cryo-EM micrograph. **c** Selected reference-free 2D class averages. **d** Gold-standard Fourier correlation curves of 3D reconstructions. **e** Posterior precision directional distributions of all particles used in the final 3D reconstruction generated by cryoSPARC. **f** The density map colored according to the local resolution estimation using cryoSPARC.

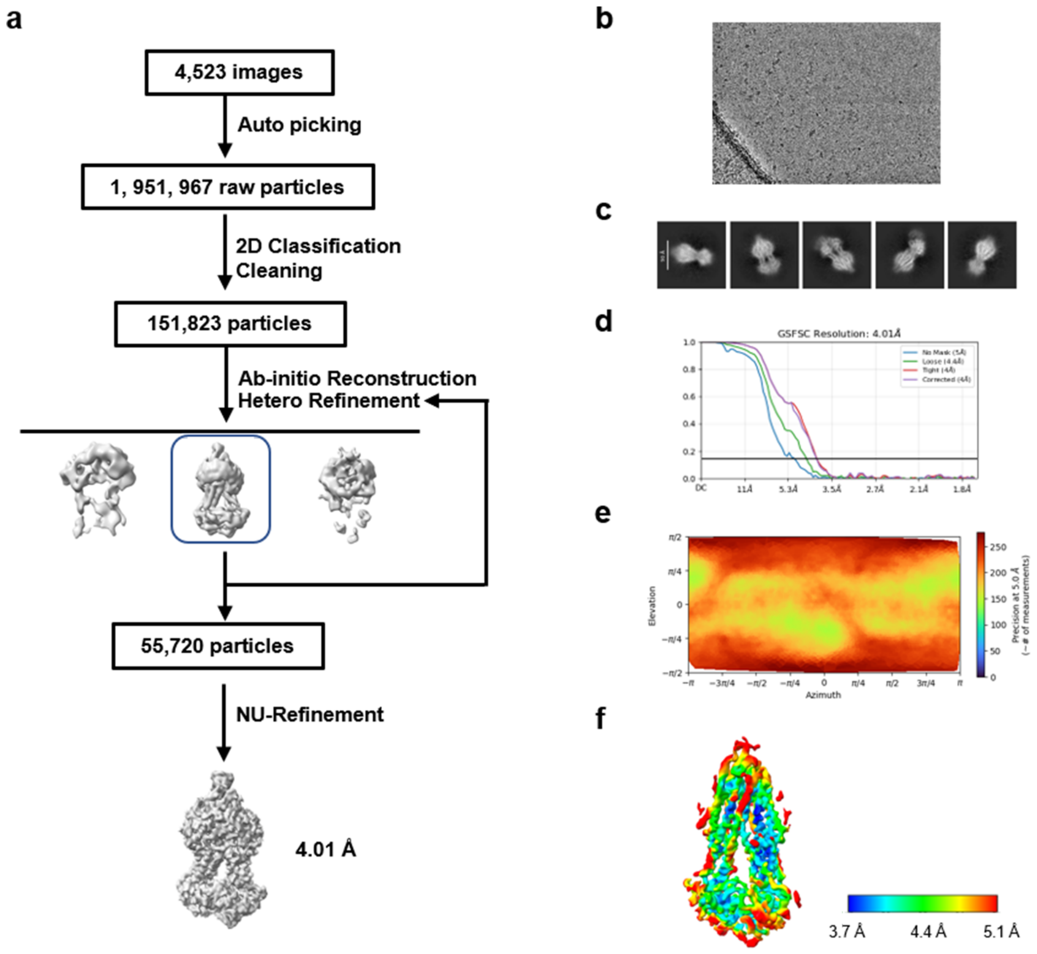

Supplementary Fig. 9 Cryo-EM data processing of *Ms*Rv1273c/72c in the ADP-bound IF$^{asym\text{-}3}$ (peptidisc) state (ADP 4 °C).

a Flow chart for the processing of the cryo-EM data. **b** Representative cryo-EM micrograph. **c** Selected reference-free 2D class averages. **d** Gold-standard Fourier correlation curves of 3D reconstructions. **e** Posterior precision directional distributions of all particles used in the final 3D reconstruction generated by cryoSPARC. **f** The density map colored according to the local resolution estimation using cryoSPARC.

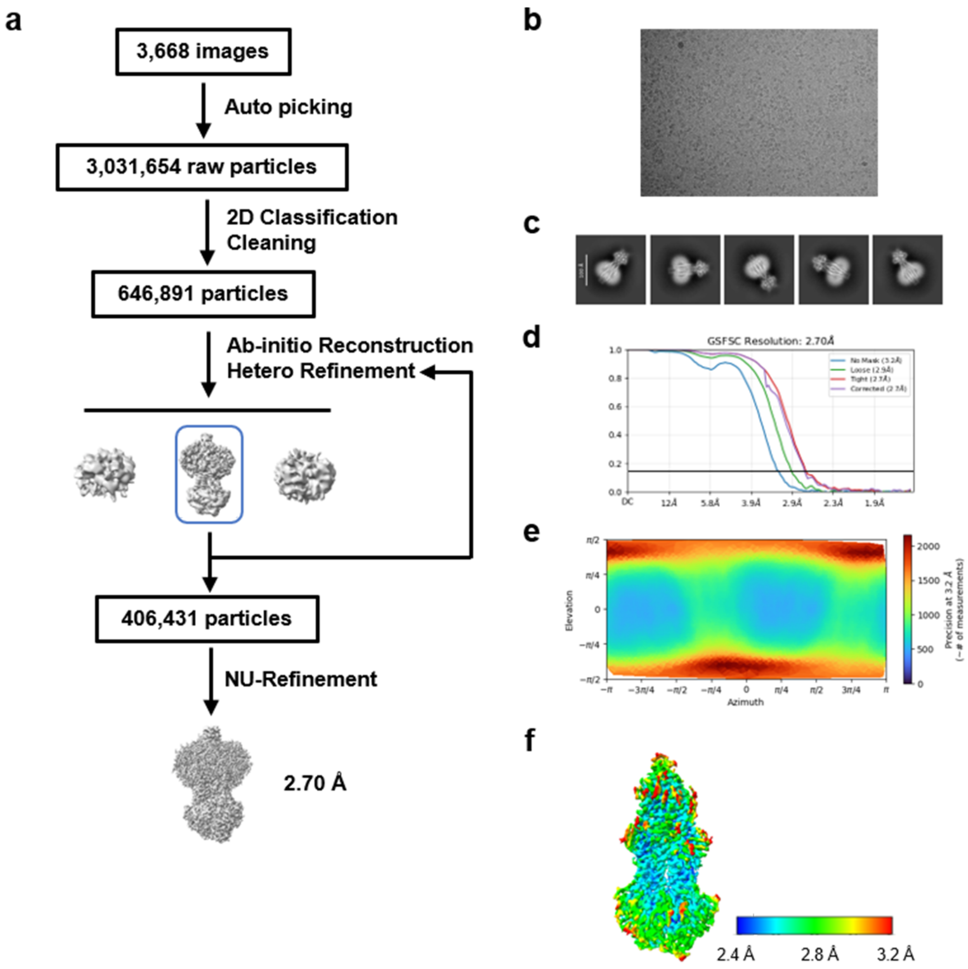

Supplementary Fig. 10 Cryo-EM data processing of *Ms*Rv1273c/72c in the ATP|ADP+Vi-bound Occ (Vi) state.

a Flow chart for the processing of the cryo-EM data. **b** Representative cryo-EM micrograph. **c** Selected reference-free 2D class averages. **d** Gold-standard Fourier correlation curves of 3D reconstructions. **e** Posterior precision directional distributions of all particles used in the final 3D reconstruction generated by cryoSPARC. **f** The density map colored according to the local resolution estimation using cryoSPARC.

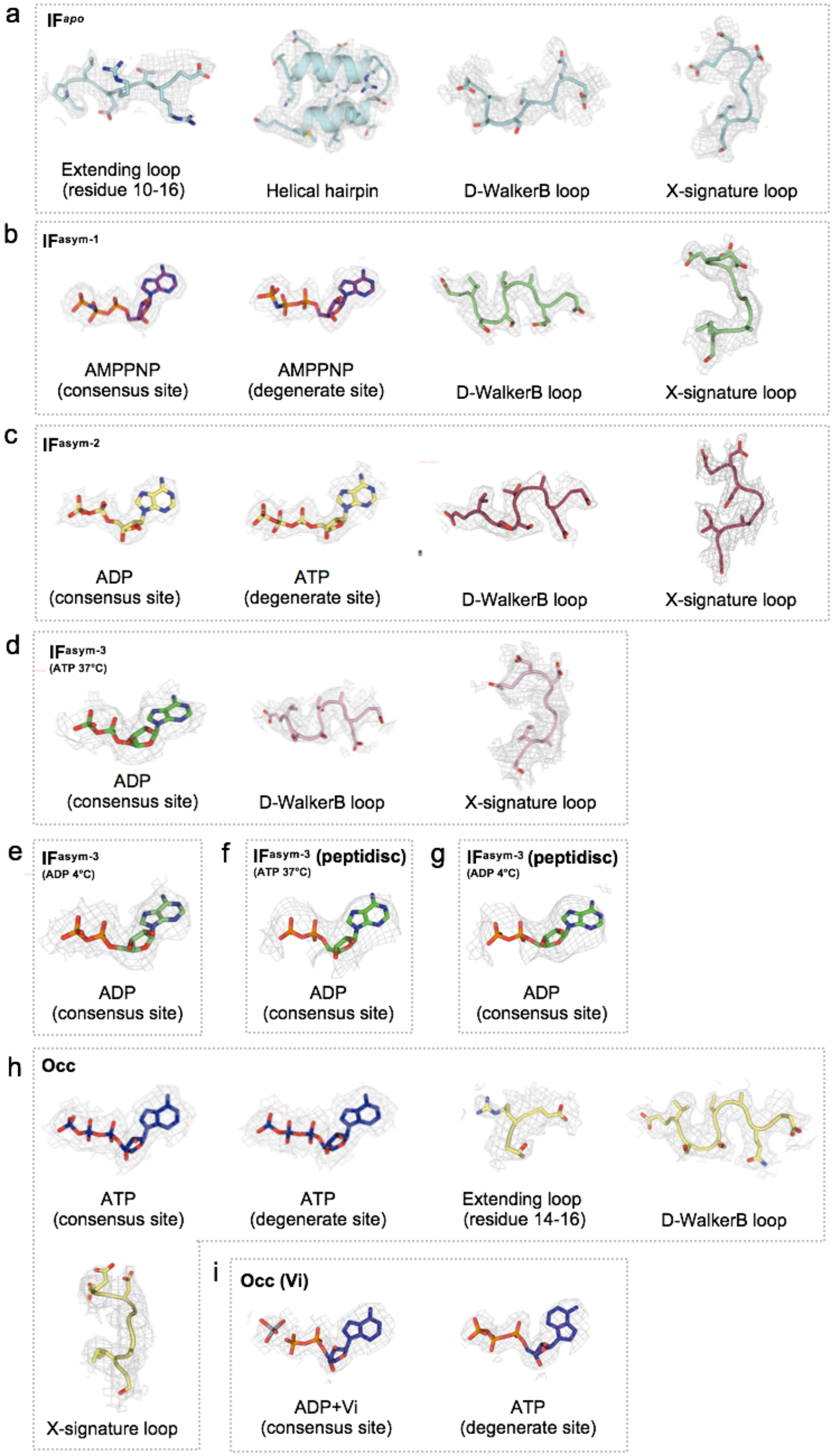

a
IFapo
Extending loop
(residue 10-16)
Helical hairpin
D-WalkerB loop
X-signature loop
b
IFasym-1
AMPPNP
(consensus site)
AMPPNP
(degenerate site)
D-WalkerB loop
X-signature loop
c
IFasym-2
ADP
(consensus site)
ATP
(degenerate site)
D-WalkerB loop
X-signature loop
d
IFasym-3
(ATP 37°C)
ADP
(consensus site)
D-WalkerB loop
X-signature loop
e
IFasym-3
(ADP 4°C)
ADP
(consensus site)
f
IFasym-3 (peptidisc)
(ATP 37°C)
ADP
(consensus site)
g
IFasym-3 (peptidisc)
(ADP 4°C)
ADP
(consensus site)
h
Occ
ATP
(consensus site)
ATP
(degenerate site)
Extending loop
(residue 14-16)
D-WalkerB loop
X-signature loop
i
Occ (Vi)
ADP+Vi
(consensus site)
ATP
(degenerate site)

Supplementary Fig. 11 Cryo-EM maps of local structures in different states of *Ms*Rv1273c/72c.

a Cryo-EM maps of the extending loop (threshold 0.27), helical hairpin (threshold 0.27), D-WalkerB loop (threshold 0.2) and X-signature motif (threshold 0.27) in the **IF**^{apo} state. **b** Cryo-EM map of AMPPNP at degenerate and consensus NBS (threshold 0.29), D-WalkerB loop (threshold 0.22) and X-signature motif (threshold 0.22) in the **IF**$^{\text{asym-1}}$ state. **c** Cryo-EM map of ADP at degenerate NBS (threshold 0.30), ATP at consensus NBS (threshold 0.30), D-WalkerB loop (threshold 0.30) and X-signature motif (threshold 0.20) in the **IF**$^{\text{asym-2}}$ state. **d** Cryo-EM map of ADP at consensus NBS (threshold 0.18), D-WalkerB loop (threshold 0.22) and X-signature motif (threshold 0.16) in the **IF**$^{\text{asym-3}}$ state (ATP 37°C). **e** Cryo-EM map of ADP at consensus NBS (threshold 0.24) in the **IF**$^{\text{asym-3}}$ state (ADP 4°C). **f** Cryo-EM map of ADP at consensus NBS (threshold 0.20) in the **IF**$^{\text{asym-3}}$ **(peptidisc)** state (ATP 37°C). **g** Cryo-EM map of ADP at consensus NBS (threshold 0.22) in the **IF**$^{\text{asym-3}}$ **(peptidisc)** state (ADP 4°C). **h** Cryo-EM map of ATP at degenerate and consensus NBS (threshold 0.25), extending loop (threshold 0.22), D-WalkerB loop (threshold 0.25) and X-signature motif (threshold 0.25) in the **Occ** state. **i** Cryo-EM map of ATP at degenerate NBS (threshold 0.34) and ADP+Vi at consensus NBS (threshold 0.34) in the **Occ (Vi)** state.

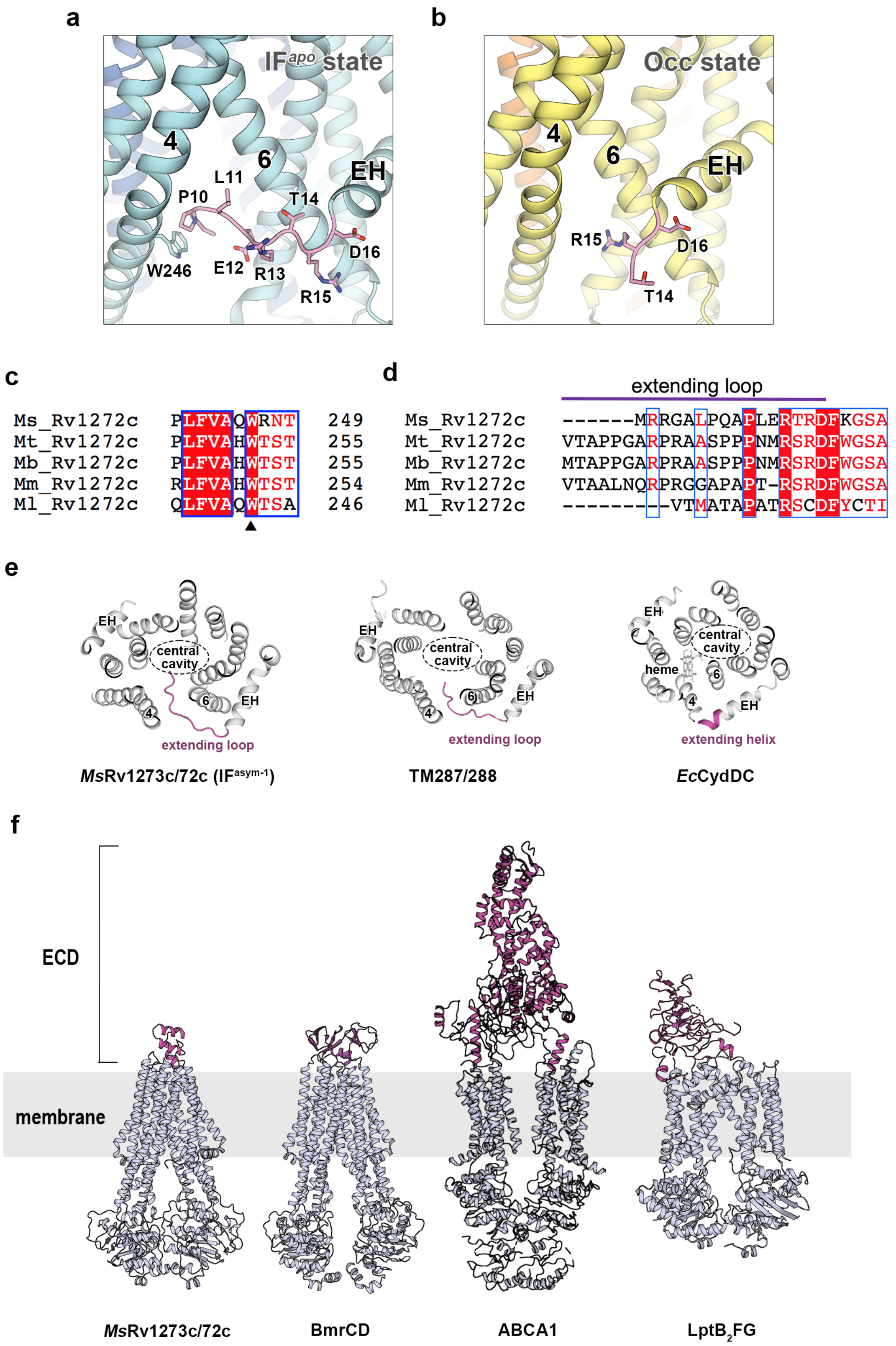

Supplementary Fig. 12 Analysis of the extending loop and ECD of *Ms*Rv1272c.

a The conformation of the extending loop (pink) in the **IF**^{apo} structure. The residues of the extending loop and interacting residue Trp246 are shown as sticks. **b** The

conformation of the extending loop (pink) in the **Occ** structure. Only the last three residues are modelled due to flexibility elsewhere. **c** Sequence alignment of TM4 near the residue Trp246 (marked with ▲) of *Ms*Rv1272c with different homologs from *Mycobacterium smegmatis* (Ms), *Mycobacterium tuberculosis* (Mt), *Mycobacterium bovis* (Mb), *Mycobacterium marinum* (Mm) and *Mycobacterium leprae* (Ml). **d** Sequence alignment of the extending loop in different Rv1272c homologs. **e** Comparison of the extending loop (magenta color) in *Ms*Rv1273c/72c in the **IF**$^{\mathbf{asym\text{-}1}}$ state with N-terminal structure (magenta color) extended from EH in other ABC transporters. The clipped views of the TMDs are presented looking from the periplasm. The central cavity is indicated by the black dashed circle. **f** Structural comparison of ECDs (magenta color) between *Ms*Rv1273c/72c and other ABC transporters.

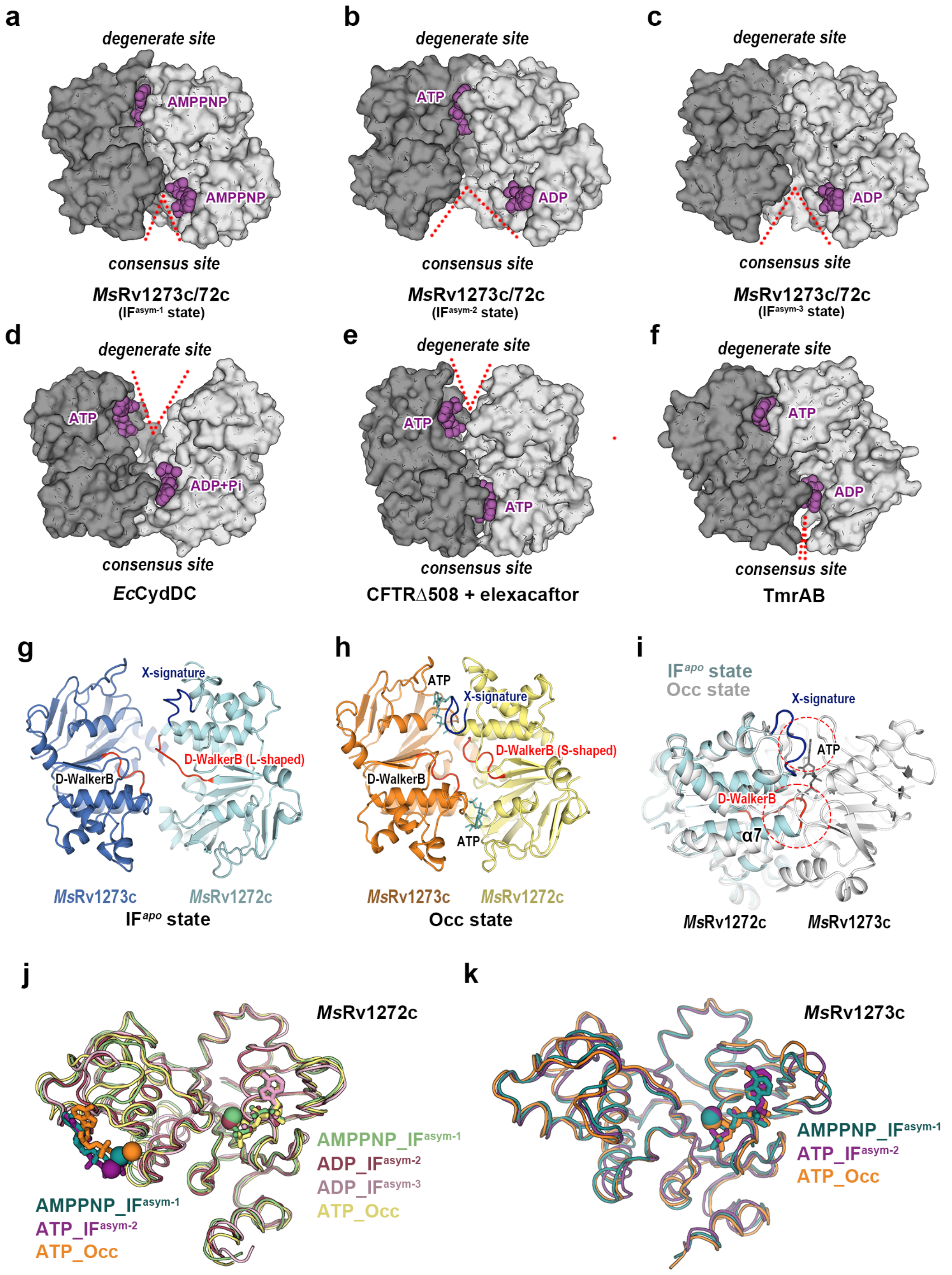

Supplementary Fig. 13 Analysis of the NBDs of *Ms*Rv1273c/72c.

a The asymmetric NBD dimers of *Ms*Rv1273c/72c in the **IF$^{\text{asym-1}}$** state. The NBDs are shown as surface models and nucleotides are shown as spheres. The size of the crack between NBDs in the partial dimer is indicated by the angle between two dashed lines. **b** The asymmetric NBD dimers of *Ms*Rv1273c/72c in the **IF$^{\text{asym-2}}$** state. **c** The asymmetric NBD dimers of *Ms*Rv1273c/72c in the **IF$^{\text{asym-3}}$** state. **d** The asymmetric

NBD dimers of *Ec*CydDC (PDB code: 7ZDA and 7ZDK). **e** The asymmetric NBD dimers of CFTR *Δ508* complexed with elexacaftor (PDB code: 8EIG). **f** The asymmetric NBD dimers of TmrAB (PDB code: 6RAM and 6RAL). **g** The L-shaped D-WalkerB loop of *Ms*Rv1272c in the **IF**^{apo} state. D-WalkerB loop and X-signature loop are highlighted in red and blue, respectively. **h** The S-shaped D-WalkerB loop *Ms*Rv1272c in the **Occ** state. **i** The NBD of *Ms*Rv1272c in the **IF**^{apo} state (cyan) is superimposed onto the same domain of the NBD dimer in the **Occ** state (grey). The α7, D-WalkerB loop, and X-signature loop will clash with the NBD of *Ms*Rv1273c or ATP (marked with dashed circles) if their conformation is not changed. **j** Superposition of NBDs of *Ms*Rv1272c in different states. The binding mode of the nucleotides at the consensus site is consistent while they are different at the degenerate site with conformational changes in the X-signature loop. **k** Superposition of NBDs of *Ms*Rv1273c in different states. The binding mode of nucleotides at the degenerate site is different with conformational changes in the A-loop.

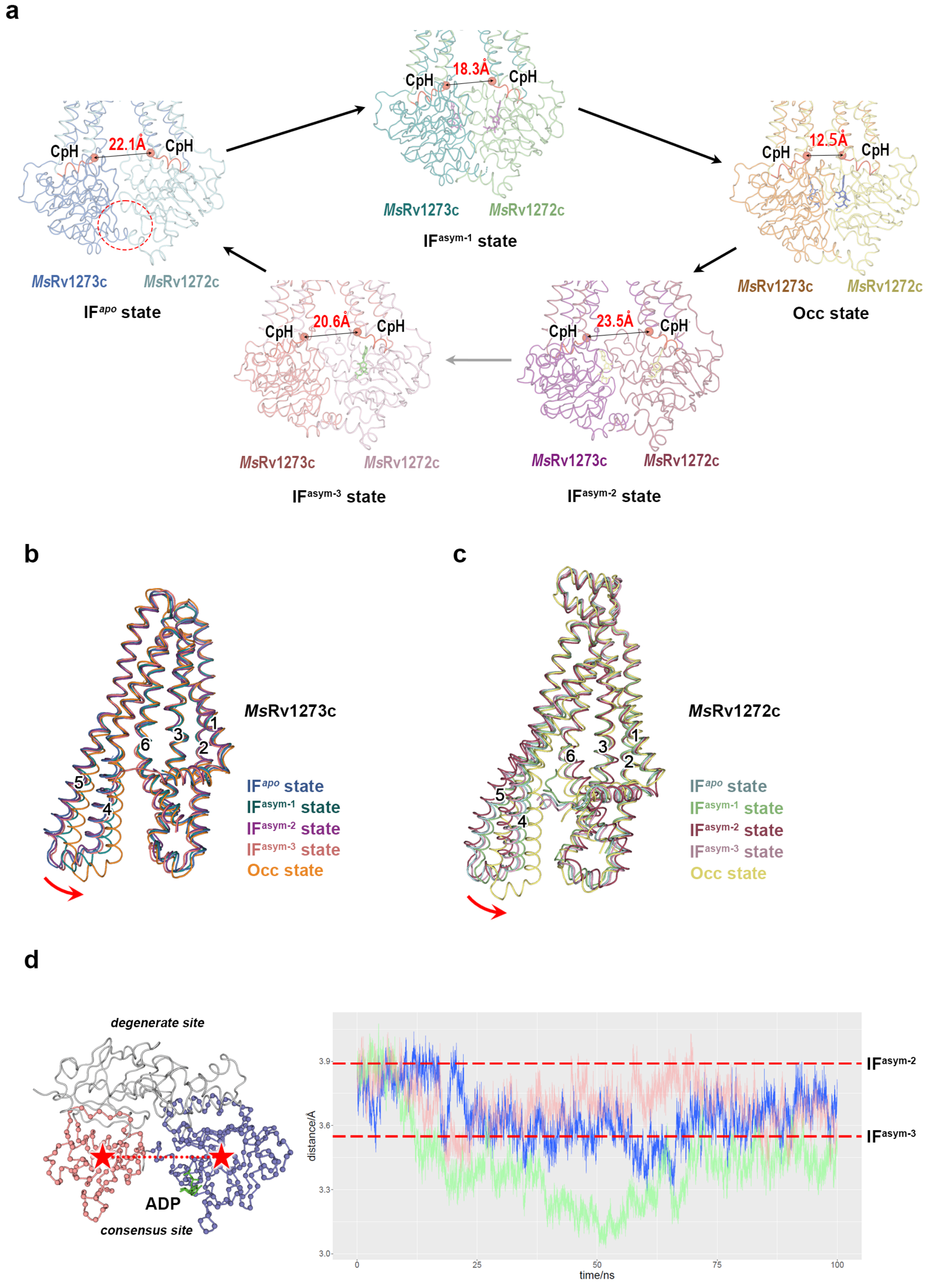

Supplementary Fig. 14 Conformational changes between different states of *Ms*Rv1273c/72c.

a The changes in distance between the two CpHs in the five structures. The distances are measured between Ser202 of *Ms*Rv1273c and Ser261 of *Ms*Rv1272c (red spheres). **b** Superposition of TMDs of *Ms*Rv1273c in the five states. The rotation of TM4-5

helices is indicated by the red arrows. **c** Superposition of TMDs of *Ms*Rv1272c in the five states. **d** The distance changes between NBDs closed to the consensus site in the MD simulation (measured between the two stars). The red star stands for the mass center of all the Cα atoms in each highlight color. The simulation starts from the **IF**$^{\mathbf{asym\text{-}2}}$ state when deleting ATP in the degenerate site. The trajectories in different color represent three independent MD runs. Configurations with distance similar to **IF**$^{\mathbf{asym\text{-}3}}$ appear in all these trajectories. The red dashed lines stand for the **IF**$^{\mathbf{asym\text{-}2}}$ and **IF**$^{\mathbf{asym\text{-}3}}$ states.

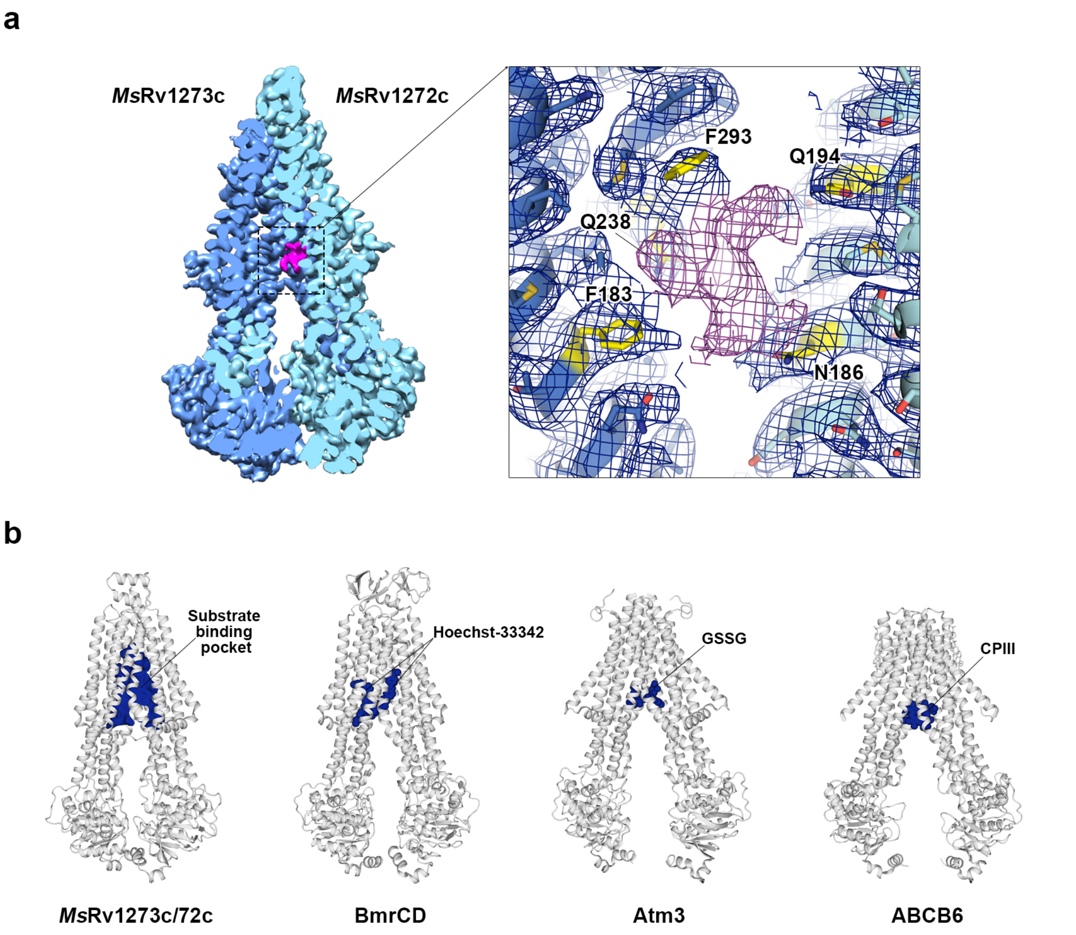

Supplementary Fig. 15 Analysis of the potential substrate binding site of *Ms*Rv1273c/72c.

a Clipped view of cryo-EM map (threshold 0.2) of *Ms*Rv1273c/72c in the **IF**^{apo} state. The additional density (magenta) in the central cavity indicates the potential substrate binding site. The right inlet is the zoom-in view of the density shown in mesh. The key surrounding residues are shown as yellow sticks. **b** Comparison of substrate binding sites between *Ms*Rv1273c/72c and other ABC transporters. The substrate binding pocket and substrates are highlighted in blue.

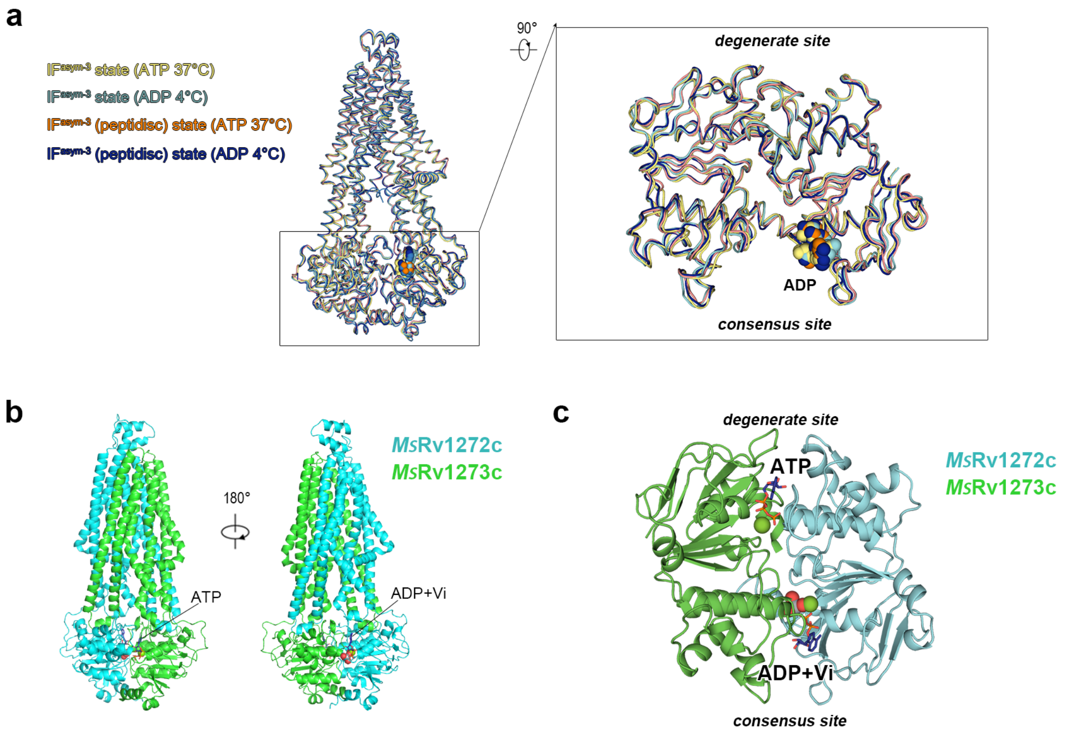

Supplementary Fig. 16 Analysis of the IF^{asym-3} and Occ states.

a Superposition of *Ms*Rv1273c/72c structures in the **IF^{asym-3}** state with different treatments. The zoom-in view shows the NBD partial dimer viewed from the periplasm. ADP is shown as spheres. **b** Structure of *Ms*Rv1273c/72c in the ATP|ADP+Vi bound **Occ (Vi)** state. **c** NBD dimer in the **Occ (Vi)** state, viewed from the periplasm.

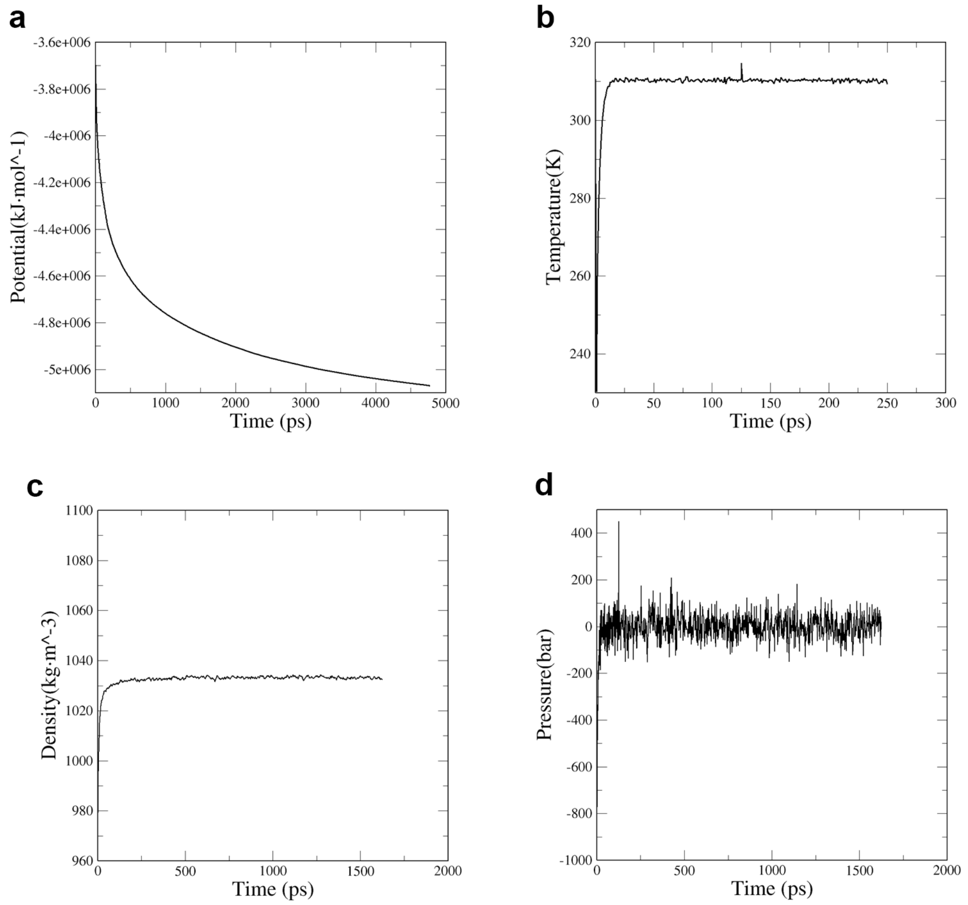

Supplementary Fig. 17 Analysis of system equilibration for MD simulation.

a Plot of potential energy change during energy minimization. **b** Plot of temperature change during NVT equilibration. **c** Plot of density change during NPT equilibration. **d** Plot of pressure change during NPT equilibration.

Supplementary Table 1 Cryo-EM data collection, refinement, and validation statistics.

	*Ms*Rv1273c/72c	*Ms*Rv1273c/72c (AMPPNP bound)	*Ms*Rv1273c/72c^{E553Q} (ATP bound)	*Ms*Rv1273c/72c (ATP|ADP bound)	*Ms*Rv1273c/72c (ADP bound)	*Ms*Rv1273c/72c (ADP bound)
State	IF^{apo}	IF^{asym-1}	Occ	IF^{asym-2}	IF^{asym-3}	IF^{asym-3}
Incubation condition	--	5 mM AMPPNP<br>4 mM $MgCl_2$<br>4 °C, 30 min	4 mM ATP<br>4 mM $MgCl_2$<br>4 °C, 30 min	10 mM ATP<br>4 mM $MgCl_2$<br>4 °C, 30 min	10 mM ATP<br>4 mM $MgCl_2$<br>37 °C, 5 min	10 mM ADP<br>4 mM $MgCl_2$<br>4 °C, 30 min
**Data collection and processing**						
Microscope	FEI Titan Krios	FEI Titan Krios	FEI Titan Krios	FEI Titan Krios	FEI Titan Krios	FEI Titan Krios
Magnification	105k ×	105k ×	105k ×	105k ×	105k ×	105k ×
Voltage (keV)	300	300	300	300	300	300
Electron exposure (e^-/Å^2)	60	60	60	60	60	60
Defocus range (μm)	-1.2 to -1.8	-1.2 to -1.8	-1.2 to -1.8	-1.2 to -1.8	-1.2 to -1.8	-1.2 to -1.8
Pixel size (Å/pixel)	0.832	0.832	0.832	0.832	0.832	0.832
Number of movies	4,863	3,206	2,564	6,663	6,176	3,411
Symmetry imposed	C1	C1	C1	C1	C1	C1
Final particle images (no.)	139,918	132,058	79,003	549,764	166,490	155,852
Map resolution (Å)	3.11	3.09	3.19	2.93	3.33	3.10
FSC threshold	0.143	0.143	0.143	0.143	0.143	0.143
**Refinement**						
Map sharpening B factor (Å^2)	112.3	117.6	120.3	153.5	152.7	134.3

Model composition						
Non-hydrogen atoms	9,062	9,199	9,127	9,130	9,119	9,119
Protein residues	1,189	1,199	1,190	1,190	1,193	1,193
Ligands	0	4	4	4	2	2
B factors ($Å^2$)						
Protein	91.85	143.80	64.15	61.89	98.38	88.69
Ligand	--	150.05	43.84	62.37	129.16	100.09
R.m.s. deviations						
Bond lengths (Å)	0.006	0.004	0.004	0.006	0.003	0.004
Bond angles (°)	0.726	0.633	0.937	1.220	0.812	0.824
Validation						
MoIProbity score	2.04	1.95	1.98	2.11	2.09	2.06
Clashscore	9.14	12.69	8.06	13.22	12.73	11.42
Poor rotamers (%)	0.21	0.42	2.94	0.73	1.04	0.31
Ramachandran plot						
Favored (%)	89.96	95.15	96.88	92.07	92.60	92.01
Allowed (%)	10.04	4.69	2.95	7.76	7.23	7.91
Disallowed (%)	0.00	0.17	0.17	0.25	0.17	0.08

Supplementary Table 1 Cryo-EM data collection, refinement, and validation statistics (continued).

	***Ms*Rv1273c/72c** (ADP bound)	***Ms*Rv1273c/72c** (ADP bound)	***Ms*Rv1273c/72c** (ATP|ADP+Vi bound)
State	**IF^{asym-3} (peptidisc)**	**IF^{asym-3} (peptidisc)**	**Occ (Vi)**
Incubation condition	10 mM ATP<br>4 mM $MgCl_2$<br>37 °C, 5 min	10 mM ADP<br>4 mM $MgCl_2$<br>4 °C, 30 min	10 mM ATP<br>4 mM $MgCl_2$<br>20 mM Na_3VO_4<br>37 °C, 5 min
**Data collection and processing**			
Microscope	FEI Titan Krios	FEI Titan Krios	FEI Titan Krios
Magnification	105k ×	105k ×	105k ×
Voltage (keV)	300	300	300
Electron exposure ($e^-/Å^2$)	60	60	60
Defocus range (μm)	-1.2 to -1.8	-1.2 to -1.8	-1.2 to -1.8
Pixel size (Å/pixel)	0.832	0.832	0.832
Number of movies	4,678	4,523	3,668
Symmetry imposed	C1	C1	C1
Final particle images (no.)	98,494	55,720	406,431
Map resolution (Å)	3.78	4.01	2.70
FSC threshold	0.143	0.143	0.143
**Refinement**			

Map sharpening B factor (Å^2)	182.0	193.8	124.2
Model composition			
Non-hydrogen atoms	9,121	9,121	9,137
Protein residues	1,193	1,193	1,191
Ligands	1	1	5
B factors (Å^2)			
Protein	91.81	153.61	59.69
Ligand	116.80	170.39	50.14
R.m.s. deviations			
Bond lengths (Å)	0.004	0.003	0.004
Bond angles (°)	1.023	0.687	0.562
Validation			
MolProbity score	2.33	1.85	1.75
Clashscore	9.90	12.08	5.93
Poor rotamers (%)	5.12	0.10	1.99
Ramachandran plot			
Favored (%)	95.96	96.22	96.80
Allowed (%)	3.78	3.62	3.03
Disallowed (%)	0.25	0.17	0.17

Table S2 The composition of the models in this study.

Structure model	Subunit	Residue built	Residue not built	Residue built (from vector)	ligand built
**IF**^{apo}	*Ms*Rv1273c	1-571	572-578	-1-0	--
	*Ms*Rv1272c	9-624	1-8, 625	--	--
**IF**$^{asym-1}$	*Ms*Rv1273c	1-572	573-578	-1-0	AMPPNP, Mg
	*Ms*Rv1272c	6-625	1-5	626-630	AMPPNP, Mg
**IF**$^{asym-2}$	*Ms*Rv1273c	1-572	573-578	-1-0	ATP, Mg
	*Ms*Rv1272c	13-625	1-12	626-628	ADP, Mg
**IF**$^{asym-3}$	*Ms*Rv1273c	1-572	573-578	-1-0	--
(ATP 37°C)	*Ms*Rv1272c	13-625	1-12	626-631	ADP, Mg
**IF**$^{asym-3}$ **(peptidisc)**	*Ms*Rv1273c	1-572	573-578	-1-0	--
(ATP 37°C)	*Ms*Rv1272c	13-625	1-12	626-631	ADP
**IF**$^{asym-3}$	*Ms*Rv1273c	1-572	573-578	-1-0	--
(ADP 4°C)	*Ms*Rv1272c	13-625	1-12	626-631	ADP, Mg
**IF**$^{asym-3}$ **(peptidisc)**	*Ms*Rv1273c	1-572	573-578	-1-0	--
(ADP 4°C)	*Ms*Rv1272c	13-625	1-12	626-631	ADP
**Occ**	*Ms*Rv1273c	1-572	573-578	-1-0	ATP, Mg
	*Ms*Rv1272c	14-625	1-13	626-629	ATP, Mg
**Occ (Vi)**	*Ms*Rv1273c	1-572	--	-2-0	ATP, Mg
	*Ms*Rv1272c	14-625	1-13	626-629	ADP, Vi, Mg
