# Supplementary material for: Structure and mechanism of a mycobacterial isoniazid efflux pump MsRv1273c/72c with a degenerate nucleotide-binding site
Source: Nat Commun. 2025 Apr 28;16:3969. doi: 10.1038/s41467-025-59300-5 (PMC12038006; doi:10.1038/s41467-025-59300-5)
Supplement: Supplementary file 2 — Description of Additional Supplementary Files [file 41467_2025_59300_MOESM2_ESM.pdf]

## Description of Additional Supplementary Files

**File Name:** Supplementary Data 1

**Description:** The sequences of primers used in this study.

**File Name:** Supplementary Data 2

**Description:** The raw LC-MS/MS spectrum used to calculate the amount of isoniazid inside the proteoliposomes after transport assay.

**File Name:** Supplementary Data 3

**Description:** The coordinate of MsRv1273c/72c structure representing the initial configuration of MD trajectories (t = 0 ns)

**File Name:** Supplementary Data 4

**Description:** The coordinate of MsRv1273c/72c structure representing the final configuration of MD trajectories (t = 100 ns)

**File Name:** Supplementary Data 5

**Description:** The coordinate of MsRv1273c/72c structure representing an intermediate configuration of MD trajectories (t = 36 ns) 6. Validation reports are provided for all the structures reported in the manuscript.
