# Supplementary material for: Structure and mechanism of a mycobacterial isoniazid efflux pump MsRv1273c/72c with a degenerate nucleotide-binding site
Source: Nat Commun. 2025 Apr 28;16:3969. doi: 10.1038/s41467-025-59300-5 (PMC12038006; doi:10.1038/s41467-025-59300-5)
Supplement: Supplementary file 8 — Reporting Summary [file 41467_2025_59300_MOESM8_ESM.pdf]

Corresponding author(s): Haitao Yang, Zihe Rao, Jun Li

Last updated by author(s): Mar 5, 2025

Reporting Summary

Nature Portfolio wishes to improve the reproducibility of the work that we publish. This form provides structure for consistency and transparency in reporting. For further information on Nature Portfolio policies, see our Editorial Policies and the Editorial Policy Checklist.

Statistics

For all statistical analyses, confirm that the following items are present in the figure legend, table legend, main text, or Methods section.

n/a	Confirmed	
☐	☒	The exact sample size (*n*) for each experimental group/condition, given as a discrete number and unit of measurement
☐	☒	A statement on whether measurements were taken from distinct samples or whether the same sample was measured repeatedly
☐	☒	The statistical test(s) used AND whether they are one- or two-sided<br>*Only common tests should be described solely by name; describe more complex techniques in the Methods section.*
☒	☐	A description of all covariates tested
☒	☐	A description of any assumptions or corrections, such as tests of normality and adjustment for multiple comparisons
☐	☒	A full description of the statistical parameters including central tendency (e.g. means) or other basic estimates (e.g. regression coefficient) AND variation (e.g. standard deviation) or associated estimates of uncertainty (e.g. confidence intervals)
☐	☒	For null hypothesis testing, the test statistic (e.g. *F*, *t*, *r*) with confidence intervals, effect sizes, degrees of freedom and *P* value noted<br>*Give P values as exact values whenever suitable.*
☒	☐	For Bayesian analysis, information on the choice of priors and Markov chain Monte Carlo settings
☒	☐	For hierarchical and complex designs, identification of the appropriate level for tests and full reporting of outcomes
☒	☐	Estimates of effect sizes (e.g. Cohen's *d*, Pearson's *r*), indicating how they were calculated

Our web collection on statistics for biologists contains articles on many of the points above.

Software and code

Policy information about availability of computer code

Data collection	Serial EM, Agilent MassHunter, Analyst
Data analysis	cryoSPARC v4.0.1; Coot; PHENIX; PyMOL; UCSF Chimera, Agilent MassHunter, Analyst, PeakView, GROMACS, R-4.3.2

For manuscripts utilizing custom algorithms or software that are central to the research but not yet described in published literature, software must be made available to editors and reviewers. We strongly encourage code deposition in a community repository (e.g. GitHub). See the Nature Portfolio guidelines for submitting code & software for further information.

Data

Policy information about availability of data

All manuscripts must include a data availability statement. This statement should provide the following information, where applicable:

- Accession codes, unique identifiers, or web links for publicly available datasets
- A description of any restrictions on data availability
- For clinical datasets or third party data, please ensure that the statement adheres to our policy

All data needed to evaluate the conclusions are present in the main text or Supplementary Materials. The cryo-EM maps of MsRv1273c/72c have been deposited in the Electron Microscopy Data Bank (EMDB) under accession codes EMD-37450 [https://www.ebi.ac.uk/pdbe/entry/emdb/EMD-37450] (IFapo state); EMD-37451 [https://www.ebi.ac.uk/pdbe/entry/emdb/EMD-37451] (AMPPNP-bound IFasym-1 state); EMD-38626 [https://www.ebi.ac.uk/pdbe/entry/emdb/EMD-38626] (ATP|ADP-bound IFasym-2 state); EMD-38627 [https://www.ebi.ac.uk/pdbe/entry/emdb/EMD-38627] (ADP-bound IFasym-3 state (ATP 37°C treated)); EMD-38628 [https://www.ebi.ac.uk/pdbe/entry/emdb/EMD-38628] (ADP-bound IFasym-3 state (ADP 4°C treated)); EMD-62611 [https://www.ebi.ac.uk/pdbe/entry/emdb/

EMD-62611] (ATP-bound Occ state); EMD-60789 [https://www.ebi.ac.uk/pdbe/entry/emdb/EMD-60789] (ADP-bound IFasym-3 (peptidisc) state (ATP 37°C treated)); EMD-60790 [https://www.ebi.ac.uk/pdbe/entry/emdb/EMD-60790] (ADP-bound IFasym-3 (peptidisc) state (ADP 4°C treated)); and EMD-60791 [https://www.ebi.ac.uk/pdbe/entry/emdb/EMD-37491] (ATP|ADP+Vi-bound Occ (Vi) state). The atomic coordinates of MsRv1273c/72c have been deposited in the Protein Data Bank (PDB) under accession codes 8WCW [https://doi.org/10.2210/pdb8WCW/pdb] (IFapo state); 8WCX [https://doi.org/10.2210/pdb8WCX/pdb] (AMPPNP-bound IFasym-1 state); 8XSR [https://doi.org/10.2210/pdb8XSR/pdb] (ATP|ADP-bound IFasym-2 state); 8XSS [https://doi.org/10.2210/pdb8XSS/pdb] (ADP-bound IFasym-3 state (ATP 37°C treated)); 8XST [https://doi.org/10.2210/pdb8XST/pdb] (ADP-bound IFasym-3 state (ADP 4°C treated)); 9KWI [https://doi.org/10.2210/pdb9KWI/pdb] (ATP-bound Occ state); 9IQE [https://doi.org/10.2210/pdb9IQE/pdb] (ADP-bound IFasym-3 (peptidisc) state (ATP 37°C treated)); 9IQF [https://doi.org/10.2210/pdb9IQF/pdb] (ADP-bound IFasym-3 (peptidisc) state (ADP 4°C treated)); and 9IQG [https://doi.org/10.2210/pdb9IQG/pdb] (ATP|ADP+Vi-bound Occ (Vi) state). The system equilibration data of MD simulation are freely available on Github (https://github.com/qifeng0000001/Rv1272_73MD). The source data underlying Fig. 1a, 1c, 1f-g, and Supplementary Fig. 1a-e, 1h are provided as a Source Data file.

Research involving human participants, their data, or biological material

Policy information about studies with human participants or human data. See also policy information about sex, gender (identity/presentation), and sexual orientation and race, ethnicity and racism.

Reporting on sex and gender	this information has not been collected
Reporting on race, ethnicity, or other socially relevant groupings	this information has not been collected
Population characteristics	this information has not been collected
Recruitment	this information has not been collected
Ethics oversight	this information has not been collected

Note that full information on the approval of the study protocol must also be provided in the manuscript.

Field-specific reporting

Please select the one below that is the best fit for your research. If you are not sure, read the appropriate sections before making your selection.

- [x] Life sciences
- [ ] Behavioural & social sciences
- [ ] Ecological, evolutionary & environmental sciences

For a reference copy of the document with all sections, see nature.com/documents/nr-reporting-summary-flat.pdf

Life sciences study design

All studies must disclose on these points even when the disclosure is negative.

Sample size	3
Data exclusions	no data were excluded
Replication	replications were successful
Randomization	this is not relevant to structural study
Blinding	this is not relevant to structural study

Reporting for specific materials, systems and methods

We require information from authors about some types of materials, experimental systems and methods used in many studies. Here, indicate whether each material, system or method listed is relevant to your study. If you are not sure if a list item applies to your research, read the appropriate section before selecting a response.

Materials & experimental systems

n/a	Involved in the study
[x]	[ ] Antibodies
[x]	[ ] Eukaryotic cell lines
[x]	[ ] Palaeontology and archaeology
[x]	[ ] Animals and other organisms
[x]	[ ] Clinical data
[x]	[ ] Dual use research of concern
[x]	[ ] Plants

Methods

n/a	Involved in the study
[x]	[ ] ChIP-seq
[x]	[ ] Flow cytometry
[x]	[ ] MRI-based neuroimaging

Plants

Seed stocks	this information has not been collected
Novel plant genotypes	this information has not been collected
Authentication	this information has not been collected
